# Using Ear Molding to Treat Congenital Auricular Deformities

**DOI:** 10.3389/fped.2021.752981

**Published:** 2021-12-16

**Authors:** Yu Chen, Wei Wang, Yue Wang, Xiang Mao

**Affiliations:** ^1^Department of Otorhinolaryngology Head and Neck Surgery, Tianjin First Central Hospital, Tianjin, China; ^2^Institute of Otolaryngology of Tianjin, Tianjin, China; ^3^Key Laboratory of Auditory Speech and Balance Medicine, Tianjin, China; ^4^Key Clinical Discipline of Tianjin (Otolaryngology), Tianjin, China; ^5^Otolaryngology Clinical Quality Control Center, Tianjin First Central Hospital, Tianjin, China

**Keywords:** ear molding, treatment duration, congenital, auricular deformation, outcome

## Abstract

**Objective:** To explore the utility of ear molding in the treatment of congenital auricular deformations.

**Study Design:** A retrospective chart/photograph review of a consecutive series of infants treated with the EarWell System from 2017 to 2020 was performed. Data on type of auricular deformity, treatment side, and auricular length and width were collected weekly for all study participants.

**Result:** A total of 173 patients (274 ears) with congenital auricular anomalies were included. The treatment duration for lop ears and Stahl's ears was shorter than for other deformations. The mean treatment EarWell duration of participants who started ear molding within 14 days of birth was shorter than that of those who started treatment more than 14 days after birth with the same ear deformation. For participants with unilateral ear deformities, the length and width of both the affected and healthy ears increased over the course of treatment, equalizing after 3 weeks. For participants with bilateral ear deformities, the length and width of both ears increased rapidly over the first 3 weeks of treatment, and the length and width of both ears gradually equalized after treatment.

**Conclusion:** Ear molding is an important intervention for treating congenital auricular deformations, and can increase auricular length and width. Early identification and initiation of treatment is crucial in the management of congenital auricular deformation.

## Introduction

Congenital auricle abnormalities are classified into two major categories: malformations and deformations. Auricle malformations are the result of an error in embryologic development and are characterized by the partial absence of the skin and/or cartilage. This results in an underdeveloped pinna that requires auricular reconstruction. Auricle deformations are characterized by a fully developed pinna without missing skin or cartilage (1, 2). Ear molding during the neonatal period offers a window of opportunity for correcting auricular deformities and less severe malformations. By intervening during the newborn period, psychosocial morbidity, pain, and surgical correction costs are avoided (3–6). This study observed and recorded the treatment duration and effect of neonatal auricle reconstruction with ear molding to provide an effective example of non-invasive clinical treatment.

## Patients and Methods

The auricular deformation was diagnosed during the initial consultation. Clinical photographic documentation was obtained before, during, and after treatment. If the ear was amenable to molding, the benefits, risks, and alternatives of the procedure were discussed with the parents.

A retrospective review of a consecutive series of infants treated with the EarWell System from 2017 to 2020 was performed. Demographic and clinical data that were collected included age, adjusted age at the time of the initial treatment, a family history of ear anomalies, pretreatment deformation or malformation type (Table 1), and physiognomic ear length and breadth. Ear length is the distance from the superaurale to the subaurale. Ear breadth is the distance from the praeaurale to the postaurale. Treatment duration was defined as the time from treatment start until the auricle shape normalized. The treatment was continued for a further 2 weeks after the anomaly was corrected.

**Table 1:** Ear anomaly classification.

**Ear anomalies**	**Characteristics**
Prominent ear	Auricle inclines forward, cranial ear angle increases, and the auricle is large and flat. The normal anatomic morphology of the auricle and anti-auricle is unknown
Cryptotia ear	The upper pole of the auricle is buried under the temporal subcutaneous tissues
Stahl′s ear	The superior auricle is flat and has an abnormal bulge
Cup ear	The auricle length becomes shorter, the triangular fossa become narrower but do not disappear, and the shape of the supine position is like a cup
Lop ear	The upper part of the auricle is pendulous
Conchal crus	The auricular foot is abnormally raised in the auricular cavity
Helical rim deformity	The ear rim does not curl, and the ear wheel is flat or not present
Constricted ear	The length of the auricle becomes shorter and the ring shrinks
Mix ear deformation	Contains 2 or more deformities
Grade I microtia	The auricle is slightly smaller but its shape is not significantly altered

A total of 274 newborn ear anomalies (173 patients) were treated with the EarWell System. The mean age for starting ear molding with the EarWell System was 15 days (3 days−3 years) (Table 2).

**Table 2:** Patient characteristics.

		**Case number**	**(%)**
Sex	male	93	53.8
	female	80	46.2
Side	uniaural	72	41.6
	biaural	101	58.4
Initial treatment age	≤1w	37	21
	1–2w	49	28
	2–3w	19	11
	3–4w	15	9
	4–8w	23	13
	8–12w	18	11
	≥12w	12	7

## Statistics

SPSS 22.0 (IBM Corp., Armonk, NY, USA) and Stata version 16.0 (Stata Corp., College Station, TX, USA) were used for data analysis. The treatment cycles for different malformations were compared. The 14-day treatment cycle of different types of malformations and the qualitative index of the variation in the length and width of the treatment of mono-ear and binaural malformations were described by percentage. Quantitative data were described by x ± s. An independent sample *t*-test was used to compare normally distributed groups, and the rank-sum test was used to compare abnormally distributed groups.

## Results

### Treatment Duration for Different Deformities

**Figure 1:**
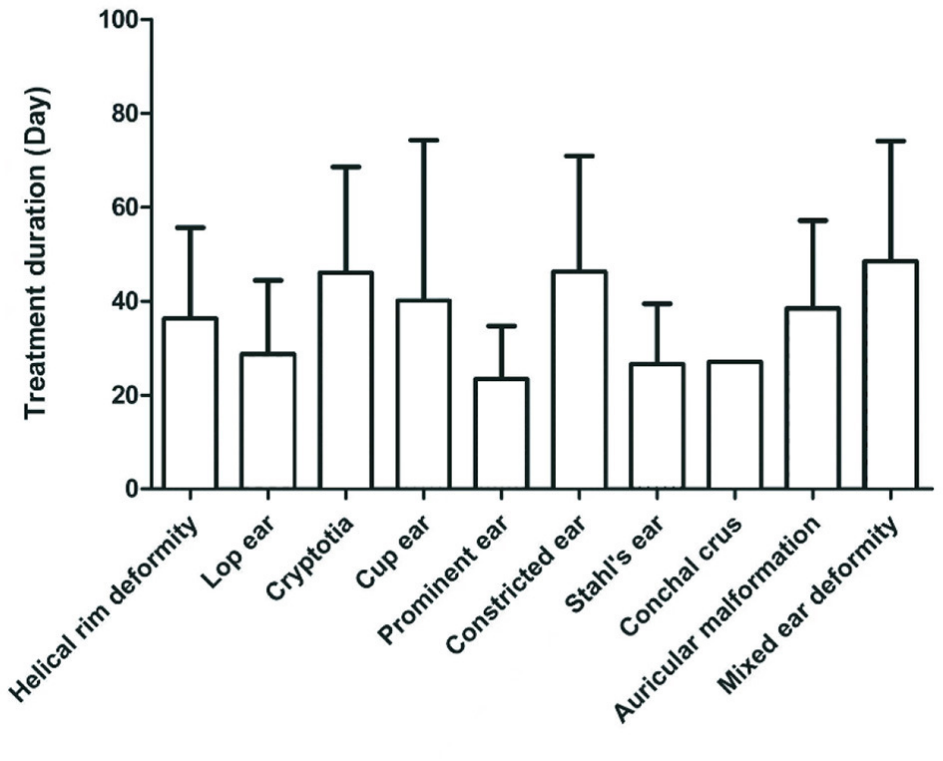
Treatment cycles of different malformation types.

Few Conchal crus cases were included in this study. Except for the Conchal crus, different deformities had significantly different treatment periods (*P* < 0.05). Treatment periods for lop ears and Stahl's ear were shorter than those of cup ears, mixed ear deformities, ringed retracted ears, cryptotias, and helical rim deformities (Figure 1).

### Duration of Treatment at Different Start Ages

The treatment durations of patients who started treatment when they were older than 14 days old vs. <14 days old were compared. Eighty-seven patients had treatment initiated when they were older 14 days old, with a mean treatment duration of 40.29 ± 23.66 days. A total of 86 patients had treatment initiated within 14 days of birth, with a mean treatment duration of (35.88 ± 21.87) days. This difference was statistically significant (*t* = 1.27, *P* < 0.05).

When comparing patients with the same type of deformation, the treatment duration of patients younger than 14 days was shorter than that of patients older than 14 days. Notably, the initial treatment of conchal crus (*n* = 1 ear), grade I microtias (*n* = 3 ears), and prominent ears (*n* = 3 ears) were not included (Table 3).

**Table 3:** The treatment cycles of different malformations in different age groups (x ± s, days).

** 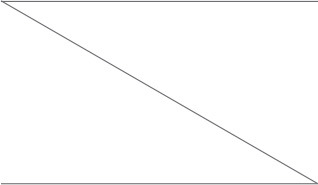 **	**Total number of ears**	**Initial treatment within 14 days**		**Initial treatment over 14 days**	
		**Number of ears**	**Treatment cycles (days)**	**Number of ears**	**Treatment cycles (days)**
Cryptotia	20	15	14.23 ± 10.91	5	44.06 ± 21.91
Lop ear	50	38	27.11 ± 13.91	12	30.47 ± 17.79
Helical rim deformity	111	70	36.11 ± 20.68	41	36.94 ± 18.27
Cup ear	25	12	37.40 ± 33.43	13	43.57 ± 41.42.
Constricted ear	27	18	46.77 ± 26.95	9	54.62 ± 23.66
Stahl's ear	17	12	24.40 ± 14.75	5	31.75 ± 4.57
Mixed ear deformity	17	8	44.11 ± 23.88	9	58.12 ± 30.65

### Length and Width of Unilateral and Bilateral Auricular Deformities

**Figure 2:**
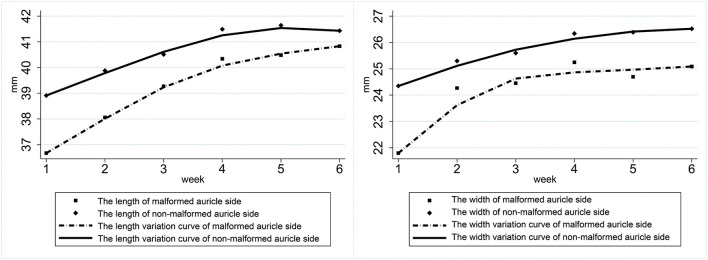
Variation in the length and width of both ears of participants with a unilateral ear deformity.

In participants with a unilateral ear deformity, both the deformed and contralateral normal ear lengths significantly increased over the first 1–3 weeks of treatment, and the difference between the ears gradually decreased and equalized. The affected ear's width increased rapidly during the first 1–2 weeks, reached peak change during the third week, and then tended to stabilize (Figure 2).

**Figure 3:**
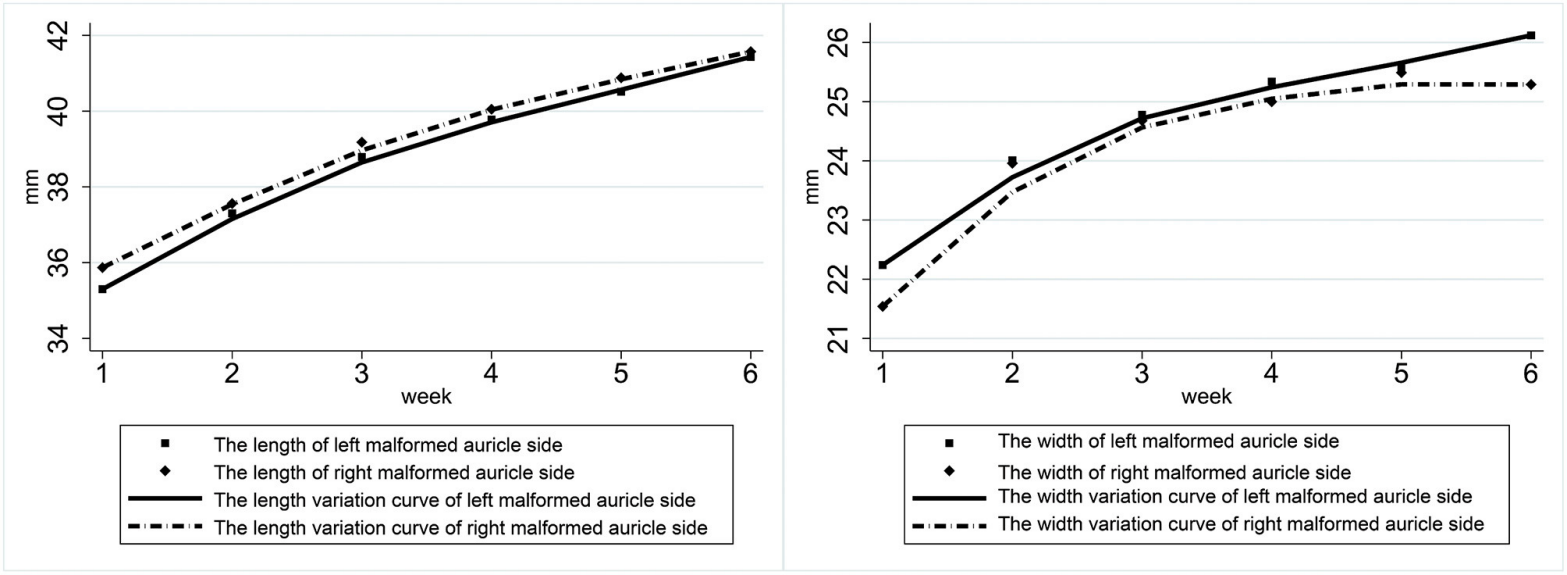
Variation in the length and width of the ears of patients with bilateral ear deformities.

In patients with bilateral ear deformities, the gap between the ears in both length and width gradually decreased during the treatment cycle. Both ears' length increased rapidly during the first 3 weeks of treatment, while the length of both ears tended to be the same after treatment. The first 3 weeks of treatment also showed a rapid increase in ear width (Figure 3).

## Discussion

The incidence of neonatal auricular malformations is reported to be 55.2–57.5% (7). As the traditional misconception that newborn auricle deformities should be observed and normalize with age, the optimal treatment period has previously been delayed.

The causes of congenital auricle malformations can be divided into genetic factors (25%), environmental factors (10%), and interaction between genetic factors (65%). Congenital absence of important anatomical structures of the ear may occur during the fifth to the ninth week of pregnancy if the embryo is poorly developed. If auricle cartilage development is abnormal during the late stage of embryogenesis, auricle morphologic deformities such as prominent ear, cup ear, and lop ear can result. The pathogenesis for these deformities may be related to the loss and division of single or multiple hillocks (8). Antenatal intrauterine and external pressures and labor canal resistance can also result in auricle morphological deformities, and the type of deformity correlates with the direction of the pressure. The internal and external auricle muscles play an important role in maintaining the auricle's normal shape. In this study, of the participants with an auricle deformity, 13 of their parents also had auricle deformities and 27 of the participants' mothers had a history of disease during pregnancy. The influence of genetic factors on auricle deformity was therefore not excluded.

The optimal time for non-invasive correction of auricle deformities is 5–7 days after birth. Residual maternal estrogen levels at birth peak within 72 h of birth and return to their baseline at 6 weeks. Estrogen can increase the content of hyaluronic acid and thus the plasticity of auricular cartilage. The auricle's plasticity greatly reduces after 6 weeks due to decreased estrogen and hyaluronic acid content in the child's blood circulation (9–11). Within 1 week of birth, patients have a 30% chance of self-healing. Appropriate massage and manipulation performed by the patient's parents within 14 days of birth can improve the self-healing rate of some auricular deformities (1, 12), particularly in the case of lobed ears with mild and moderate deformities and Stahl's ears. In the present study, patients with droop ears and Stahl's ears had no significant improvement after 1 week of observation, prompting the use of non-invasive corrective techniques. Tan et al. believed that non-invasive correction within 3 months of birth had an ideal effect, and that treatment timing was closely related to the duration of the curative effects (2). Byrd et al. reported that the treatment course should be prolonged in patients older than 3 weeks of age, and that its efficacy was halved (1). In this study, participants with an initial diagnosis that were younger than 14 days-old had a short treatment period while patients over 14 days old and six patients with an age at diagnosis of >100 days had significantly longer average treatment periods (65.33 ± 24.85 days) and their ear shape was prone to rebound. These findings support a relationship between younger age at ear molding and shorter treatment durations.

Different types of auricular deformities require different treatment cycles. The treatment cycles for the lop ear and Stahl's ear were the shortest. A lop ear is characterized by the folding of the helix itself and tentacle drooping of the upper part of the auricle to cover the opposite helix's upper leg, which eventually leads to a reduction in the length of the auricle. Lop ear morphology differs greatly from that of normal ears and it is generally quite obvious to parents at an early age. Therefore, compared with other types of malformations, lop ears are diagnosed at a younger age and have a shorter treatment duration. Cryptotia ears occur when the posterior cranial sulcus becomes shallow or disappears without an obvious posterior auricular sulcus. Pulling the upper auricle outward can recreate the auricle's complete appearance, but the deformity returns after the pull is released. Patients with severe cryptotia suffer from severe skin shortage of the auricle and chondrodysplasia of the upper auricle. The cryptotia ear is usually an upper auricle deformity that is difficult for parents to detect, and the treatment duration is longer when it is diagnosed at an older age (13–15). During the first stage of treatment the upper edge of the auricle embedded under the scalp is pulled out (16–18), which takes about 2 weeks. During the second stage the auricle is shaped by applying a lower frame, which involves a relatively long treatment duration. Early diagnosis of the prominent ear is the most difficult and easily neglected, and Byrd believed that the diagnosis could only be made when the distance between the middle helium and the lateral cranial wall was >1 cm (1). As the cranio-auricular angle gradually enlarges, the optimal treatment period is often missed. In this study, three prominent ears were observed in patients older than 14 days after birth, all of whom required a treatment period longer than 4 weeks. Enlargement of the cranio-auricular angle is due to the excessive growth of the auricular cartilage (19), which has a high probability of rebound and a long treatment cycle. It is therefore important to increase the publicity about prominent ears, which may improve their early detection and early treatment.

**Figure 4:**
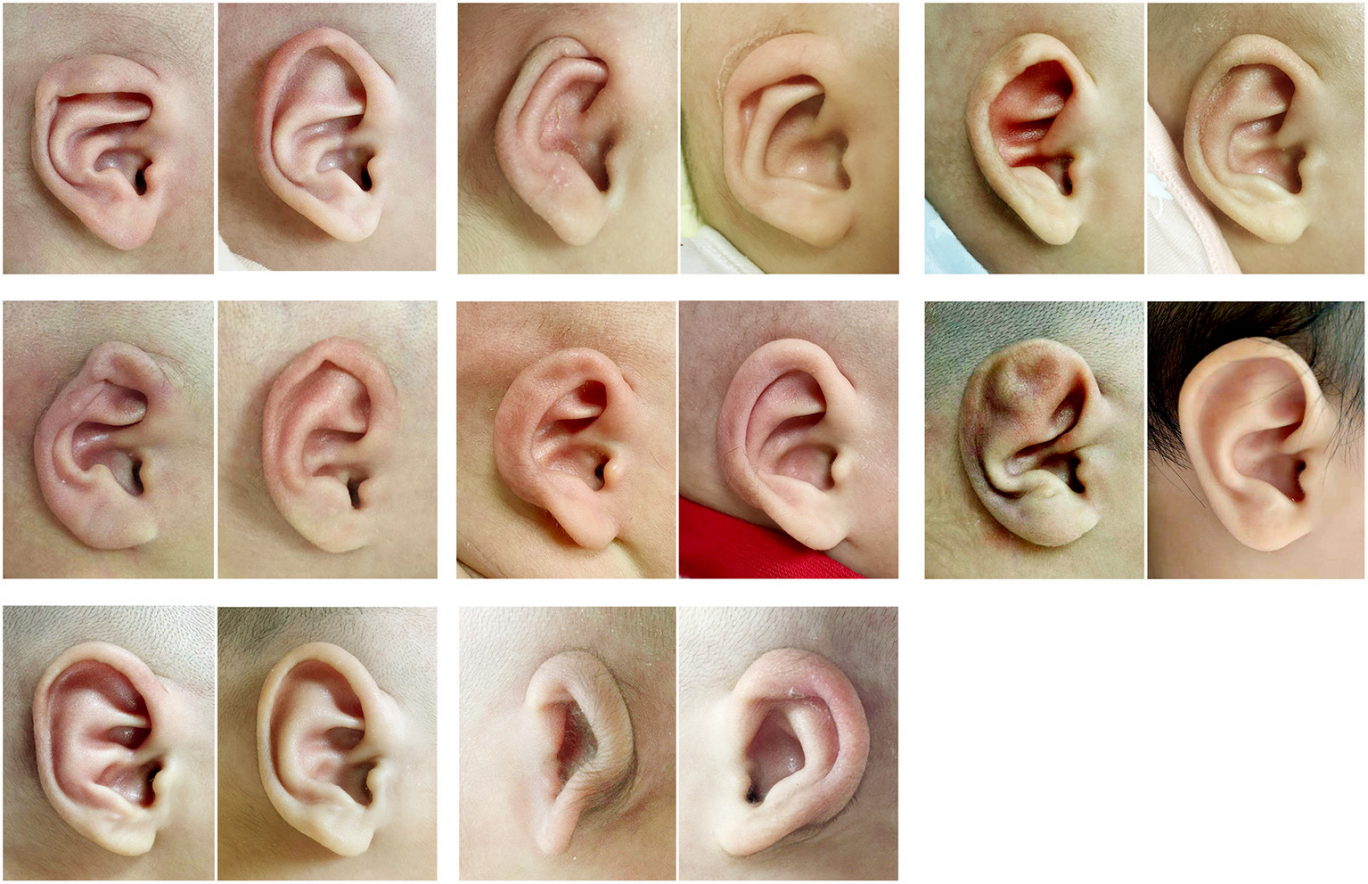
Pretreatment (left in each pair) and posttreatment (right in each pair) photographs of a child with a lop ear (top left), cryptotia (top middle), cup ear (top right), helical rim deformity (middle left), constricted ear (middle middle), Stahl's ear (middle right), conchal crus (lower left), and a grade I microtia (lower middle).

Ear molding has a supportive effect on the auricle. The physiognomic ear length and breadth are main measuring index of auricle. In this study, we measured the physiognomic ear length and breadth weekly. The result showed that in patients with unilateral ear deformities the length and width of both the affected ear and the healthy ear increased over the course of treatment. Both ear lengths increased significantly over 1–3 weeks, the differences between the ears gradually decreased and equivalency was achieved. The affected ear's width also increased rapidly in 1–2 weeks, reached its maximum during the third week, and then grow steadily. In participants with bilateral ear deformities, the length and width of both ears gradually increased over the course of treatment, and the gap between the ear gradually decreased. The length of both ears increased rapidly during the first 3 weeks of treatment and tended to be the same after treatment (Figure 4). Ear width also rapidly increased during the first 3 weeks of treatment.

## Conclusion

Ear molding is non-invasive, has few complications and is low cost. It is an important method for treating neonatal auricle malformations and should be widely promoted clinically. Early identification and prompt initiation of treatment are crucial to its success.

## Data Availability Statement

The original contributions presented in the study are included in the article/Supplementary Material, further inquiries can be directed to the corresponding author/s.

## Ethics Statement

The studies involving human participants were reviewed and approved by Tianjin First Central Hospital. Written informed consent to participate in this study was provided by the participants' legal guardian/next of kin.

## Author Contributions

WW and YW organized the database. XM performed the statistical analysis. YC wrote the first draft of the manuscript. WW, YW, YC, and XM wrote sections of the manuscript. All authors contributed to conception, design of the study and manuscript revision, read, and approved the submitted version.

## Funding

This study was supported by Key Clinical Discipline of Tianjin; National Natural Science Foundation of China (81971698) and Tianjin Natural Science Foundation (19JCYBJC27200). The authors declare that they have no competing interests.

## Conflict of Interest

The authors declare that the research was conducted in the absence of any commercial or financial relationships that could be construed as a potential conflict of interest.

## Publisher's Note

All claims expressed in this article are solely those of the authors and do not necessarily represent those of their affiliated organizations, or those of the publisher, the editors and the reviewers. Any product that may be evaluated in this article, or claim that may be made by its manufacturer, is not guaranteed or endorsed by the publisher.

## Supplementary Material

Supplementary Figure 1 The physiognomic ear length and breadth were measured. The length is the straight-line distance between the highest point of the upper margin of the auricle and the lowest point of the earlobe when the head is at Frankfurt level. The breadth is the straight-line distance between the point at which the upper edge of the auricle is attached to the cephalic side and the point at which the posterior edge of the auricle protrudes most backward when the head is at Frankfurt level.
