# Supplementary figures and images for: Using Ear Molding to Treat Congenital Auricular Deformities
Source: Front Pediatr. 2021 Dec 16;9:752981. doi: 10.3389/fped.2021.752981 (PMC8717866; doi:10.3389/fped.2021.752981)

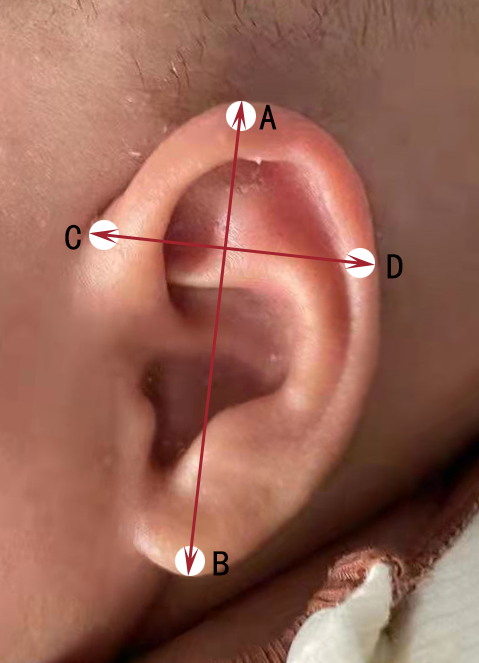

Supplement: Supplementary Figure 1 — The physiognomic ear length and breadth were measured. The length is the straight-line distance between the highest point of the upper margin of the auricle and the lowest point of the earlobe when the head is at Frankfurt level. The breadth is the straight-line distance between the point at which the upper edge of the auricle is attached to the cephalic side and the point at which the posterior edge of the auricle protrudes most backward when the head is at Frankfurt level. [file Image_1.TIF]
